# Programmed cell death-1: from a T-cell immune checkpoint to a regulator of Natural Killer cell biology

**DOI:** 10.3389/fcell.2026.1924522

**Published:** 2026-08-19

**Authors:** Fabiana De Franco, Marco Greppi, Valentina Obino, Federico Rebaudi, Rayan Goda, Irene Caffa, Matteo Bozzo, Ombretta Melaiu, Enrico Munari, Valerio Gaetano Vellone, Camilla Jandus, Domenico Mavilio, Simona Candiani, Silvia Pesce, Emanuela Marcenaro

**Affiliations:** 1 Department of Experimental Medicine, DIMES, University of Genoa, Genoa, Italy; 2 Department of Internal Medicine and Medical Specialties, University of Genoa, Genoa, Italy; 3 AOM - IRCCS Ospedale Policlinico San Martino, Genoa, Italy; 4 Department of Earth, Environmental and Life Sciences, DISTAV, University of Genoa, Genoa, Italy; 5 Department of Clinical Sciences and Translational Medicine, University of Rome Tor Vergata, Rome, Italy; 6 Laboratory of Medical Genetics, Translational Cytogenomics Research Unit, Bambino Gesù Children Hospital, IRCCS, Rome, Italy; 7 Pathology Unit, Verona University Hospital Trust, Verona, Italy; 8 Department of Integrated Surgical and Diagnostic Sciences, University of Genoa, Genoa, Italy; 9 Pathology Unit, IRCCS Istituto Giannina Gaslini, Genoa, Italy; 10 Department of Pathology and Immunology, Faculty of Medicine, University of Geneva, Geneva, Switzerland; 11 Ludwig Institute for Cancer Research, Lausanne Branch, Lausanne, Switzerland; 12 Geneva Center for Inflammation Research, Geneva, Switzerland; 13 Translational Research Centre in Onco-Haematology (CRTOH), Geneva, Switzerland; 14 Institute of Genetics and Genomics of Geneva (iGE3), University of Geneva, Geneva, Switzerland; 15 Laboratory of Clinical and Experimental Immunology, IRCCS Humanitas Research Hospital, Department of Medical Biotechnology and Translational Medicine, University of Milan, Milan, Italy

**Keywords:** cancer immunotherapy, immune checkpoint, Natural Killer (NK) cells, programmed cell death 1 (PD-1), Tumor Microenviroenment (TME)

## Abstract

Programmed cell death protein 1 (PD-1, CD279) is a pivotal inhibitory immune checkpoint receptor that plays a central role in maintaining immune homeostasis and peripheral tolerance. Originally characterized as a negative regulator of T-cell activation, PD-1 limits excessive immune responses and prevents autoimmunity, while its sustained expression under conditions of chronic antigen stimulation contributes to T-cell dysfunction and exhaustion. The discovery that blockade of the PD-1 pathway can restore anti-tumor immunity has revolutionized cancer therapy and established immune checkpoint inhibition as a cornerstone of modern oncology. Although PD-1 has traditionally been viewed as a key regulator of adaptive immunity, accumulating evidence indicates that its biological functions extend beyond T cells. In recent years, PD-1 expression has been identified in several innate immune cell populations, particularly Natural Killer (NK) cells, where it has emerged as an important modulator of effector functions, cytokine production, metabolic fitness, and antitumor activity. These findings have challenged the classical view of PD-1 biology and revealed unexpected similarities between NK-cell dysfunction and the exhausted phenotype described in chronically stimulated T cells. In the tumor microenvironment, PD-1 expression on NK cells has been associated with impaired cytotoxicity and reduced immune surveillance, suggesting that NK cells may also represent relevant targets of PD-1-mediated immunosuppression. At the same time, the mechanisms regulating PD-1 expression and signaling in NK cells appear to differ, at least in part, from those operating in T lymphocytes, highlighting the complexity of this pathway across distinct immune cell subsets. In this review, we summarize the current knowledge of PD-1 biology, from its established role in T-cell regulation to its emerging functions in NK cells. We discuss the molecular mechanisms governing PD-1 expression and signaling, its contribution to immune dysfunction in cancer and chronic diseases, and the potential implications of targeting the PD-1 axis to enhance both adaptive and innate antitumor immunity.

## 1 Introduction

Programmed cell death-1 (PD-1, CD279) is a paradigmatic inhibitory immune checkpoint receptor that has fundamentally reshaped our understanding of immune regulation across both physiological and pathological conditions. Originally identified in 1992 b y Honjo and colleagues during a screen for genes associated with programmed cell death, PD-1 was described as a novel activation-induced member of the immunoglobulin superfamily (Ishida et al., 1992). Subsequent studies revealed that, rather than directly regulating cell death, PD-1 functions as a critical inhibitory receptor controlling immune responses (Iwai et al., 2002; Topalian et al., 2012; Sharma and Allison, 2015).

Genetic ablation of PD-1 in murine models resulted in the spontaneous development of autoimmune phenotypes, including lupus-like disease, autoimmune cardiomyopathy, and glomerulonephritis, thereby demonstrating its non-redundant role in the maintenance of peripheral tolerance (Nishimura et al., 1999; Keir et al., 2008). These findings positioned PD-1 as a key immune checkpoint that restrains excessive or dysregulated immune activation, and it protects tissues from immune-mediated damage, often acting in concert with other inhibitory pathways such as CTLA-4.

Conversely, in settings of chronic antigen exposure, including persistent viral infections and cancer, sustained PD-1 expression contributes to progressive immune dysfunction. In T cells, prolonged PD-1 signaling promotes the establishment of an exhausted phenotype characterized by reduced proliferative capacity, impaired cytokine production, and diminished cytotoxic function (Wherry, 2011; Pauken and Wherry, 2015). Within the tumor microenvironment (TME), malignant cells exploit this pathway by upregulating PD-L1, thereby engaging PD-1 on effector lymphocytes and suppressing antitumor immunity (Iwai et al., 2002; Topalian et al., 2012; Sharma and Allison, 2015; Blank et al., 2005; Blank and Mackensen, 2007; Hamanishi et al., 2007; Hino et al., 2010; Mu et al., 2011; Pardoll, 2012).

The therapeutic targeting of the PD-1/programmed death ligands (PD-L1/PD-L2) axis has led to a paradigm shifts in oncology. Monoclonal antibodies blocking PD-1 or its ligands have demonstrated the capacity to restore immune effector functions and induce durable tumor regression in multiple malignancies.

First-in-human clinical trials using anti-PD-1 antibodies (nivolumab, BMS-936558) demonstrated durable responses in melanoma, non-small cell lung cancer (NSCLC), and renal cell carcinoma (Topalian et al., 2012; Garon et al., 2015), leading to the first regulatory approvals in melanoma in 2014 (Weber et al., 2015; Robert et al., 2015). These seminal discoveries ultimately led to the awarding of the 2018 Nobel Prize in Physiology or Medicine to Tasuku Honjo for the discovery of PD-1 and its development as a therapeutic immune checkpoint (Huang and Chang, 2019; Wei et al., 2019).

Although PD-1 was initially characterized within the adaptive immune compartment, accumulating evidence has expanded its relevance to innate immunity, particularly Natural Killer (NK) cells. NK cells are key effectors of early antiviral and antitumor responses and have traditionally been viewed as regulated predominantly by germline-encoded activating and inhibitory receptors. However, the identification of PD-1 expression on NK cells has challenged this paradigm and revealed previously unappreciated similarities between NK-cell dysfunction and T-cell exhaustion programs.

Recent evidence demonstrated that PD-1 can be expressed on mature NK cells from healthy adult individuals, particularly in the context of prior human cytomegalovirus (HCMV) infection, where it contributes to the fine-tuning of NK-cell activation and functional adaptation (Pesce et al., 2017; Pesce et al., 2019; Del et al., 2017; Mariotti et al., 2019). Moreover, PD-1 expression has been detected in neonatal NK cells, where it plays a role in regulating NK cell maturation/licensing and preventing inappropriate activation during immune system development (Greppi et al., 2023).

In pathological conditions, including both hematological malignancies and solid tumors, PD-1 expression on NK cells has been consistently associated with impaired cytotoxicity and reduced antitumor activity (Pesce et al., 2017; Pesce et al., 2019; Benson et al., 2010; MacFarlane et al., 2014; Beldi-Ferchiou et al., 2016; Liu et al., 2017; Vari et al., 2018; Concha-Benavente et al., 2018; Trefny et al., 2020; Farhat et al., 2024; Tumino et al., 2019; Quatrini et al., 2021; Quatrini et al., 2018). Notably, accumulating evidence indicates that PD-1 is frequently co-expressed with additional inhibitory receptors, thereby contributing to the establishment of deeply dysfunctional NK-cell subsets within the TME (Rebaudi et al., 2024; Greppi et al., 2024).

Recent data has repositioned PD-1 as a pleiotropic immune regulator whose function extends well beyond T-cell biology to critically orchestrate NK cell activity across both physiological and pathological settings. In this context, the emerging paradigm of coordinated checkpoint co-expression, particularly involving PD-1 and inhibitory receptors such as NKG2A, reveals a multilayered regulatory network that enforces profound immune suppression within the TME (André et al., 2018; Greppi et al., 2025). This combinatorial inhibitory landscape not only refines our understanding of NK-cell dysfunction but also provides a strong rationale for the development of next-generation immunotherapeutic strategies based on dual or multi-checkpoint blockade. In this review, we examine the evolving biology of PD-1 and its translational relevance, with a specific emphasis on NK cells, highlighting recent advances in solid tumors where PD-1-associated regulatory circuits have emerged as important determinants of tumor immune escape.

## 2 The PD-1/PD-ls axis in its canonical T-cell context

The PD-1/PD-Ls axis constitutes a central regulatory pathway in immune homeostasis, acting as a dynamic checkpoint that calibrates immune activation and preserves peripheral tolerance. PD-1 is an inducible inhibitory receptor expressed upon antigen receptor engagement across multiple immune cell types, where its expression is tightly regulated by both transcriptional and epigenetic mechanisms (Pesce et al., 2017; Blank et al., 2019; Myers et al., 2022). Early activation-driven expression of PDCD1 is mediated by transcription factors such as NFAT, whereas sustained expression under chronic stimulation is maintained by factors including FOXO1 and stabilized through epigenetic remodeling of the PDCD1 locus (Oestreich et al., 2008; Staron et al., 2014; Youngblood et al., 2011). Conversely, lineage-defining transcription factors such as T-bet act as negative regulators of PD-1 expression on CD8^+^ T cells, thereby coupling cellular differentiation programs to immune checkpoint control (Kao et al., 2011).

The physiological relevance of PD-1 signaling in maintaining immune tolerance is underscored by both preclinical and clinical evidence. In autoimmune-prone models, disruption of PD-1 or PD-L1 signaling accelerates disease onset and severity, as demonstrated in the non-obese diabetic mouse, where this pathway restrains auto-reactive T-cell responses (Keir et al., 2006; Ansari et al., 2003). In humans, loss-of-function mutations in PDCD1 result in severe early-onset autoimmunity and immune dysregulation, while PD-L1 deficiency similarly compromises immune homeostasis (Ogishi et al., 2021; Johnson et al., 2024). Together, these findings establish the PD-1/PD-L axis as a nonredundant safeguard against immune-mediated pathology.

At the molecular level, PD-1 is a type I transmembrane glycoprotein containing an extracellular IgV-like domain and a cytoplasmic tail with two key signaling motifs, ITIM and ITSM. Upon engagement by its ligands PD-L1 or PD-L2, PD-1 undergoes phosphorylation and recruits SHP-2 phosphatase (and to a lesser extent SHP-1), resulting in attenuation of proximal signaling events downstream of antigen receptors (Keir et al., 2008; Pesce et al., 2017; Chemnitz et al., 2004; Hui et al., 2017; Patsou et al., 2020). Importantly, PD-1 signaling is spatially restricted, requiring co-localization with activating receptors at the immunological synapse, where it forms inhibitory microclusters that directly interfere with signalosome assembly (Hui et al., 2017; Yokosuka et al., 2012).

Mechanistically, PD-1 dampens immune activation by targeting key signaling nodes. It inhibits phosphorylation of CD3ζ and ZAP70, suppresses downstream PKCθ activation, and directly antagonizes PI3K activity, resulting in reduced AKT signaling and metabolic reprogramming of immune cells (Keir et al., 2008; Chemnitz et al., 2004; Sheppard et al., 2004; Parry et al., 2005; Boussiotis and Patsoukis, 2022). Notably, CD28 has emerged as a preferential target of PD-1-mediated inhibition, highlighting the importance of PD-1 in modulating co-stimulatory signaling (Hui et al., 2017). These effects collectively lead to reduced cytokine production, including IFN-γ, TNF-α, and IL-2, impaired survival due to decreased expression of anti-apoptotic molecules such as Bcl-xL, and altered differentiation programs involving transcription factors such as T-bet, EOMES, and GATA-3 (Keir et al., 2008; Wherry, 2011; Carter et al., 2002; Freeman et al., 2000).

The ligands PD-L1 and PD-L2 (Latchman et al., 2001) contribute an additional layer of regulation through their distinct expression patterns and inducibility. PD-L1 is broadly expressed across hematopoietic and non-hematopoietic tissues and is strongly upregulated by inflammatory cues, particularly interferons, *via* transcriptional programs involving IRF-1 and signaling pathways such as JAK/STAT and PI3K/AKT (Chen and Mellman, 2017; Garcia-Diaz et al., 2017). In contrast, PD-L2 expression is more restricted and primarily confined to antigen-presenting cells, where it is induced by cytokines such as IL-4, GM-CSF, and IFN-γ. The coordinated expression of PD-1 and its ligands enable context-dependent modulation of immune responses, fine-tuning the balance between activation and tolerance.

In pathological settings, including chronic infections and cancer, persistent engagement of the PD-1/PD-L axis drives the establishment of dysfunctional immune states. Sustained PD-1 expression promotes a progressive loss of effector functions and the acquisition of an exhausted phenotype, while tumor cells exploit PD-L1 upregulation to evade immune surveillance (Iwai et al., 2002; Topalian et al., 2012; Wherry, 2011; Pauken and Wherry, 2015). Importantly, the impact of PD-1 signaling extends beyond adaptive immunity, influencing multiple immune compartments and contributing to a broader immunosuppressive network within the TME.

Beyond its well-established role in proximal signaling inhibition, PD-1 has emerged as an important regulator of cellular metabolism. In T cells, PD-1 engagement suppresses glycolytic flux, impairs mitochondrial biogenesis, and promotes a shift toward a metabolically quiescent state, ultimately reducing cellular fitness and effector function (Blank et al., 2019; Patsoukis et al., 2015; Bengsch et al., 2016; Buck et al., 2017; Scharping et al., 2021).

Altogether, the PD-1/PD-L axis represents a highly coordinated and multilayered regulatory system in which receptor-intrinsic properties, ligand availability, and microenvironmental cues converge to shape immune cell fate and function. A deeper understanding of this integrated biology may provide a framework for modulating checkpoint signaling in cancer and autoimmune diseases and highlights the potential relevance of PD-1 biology beyond T cells, including its emerging roles in other immune cell subsets such as NK cells, which will be discussed in the following sections.

## 3 PD-1 emergence within the NK-Cell checkpoint landscape

**FIGURE 1:**
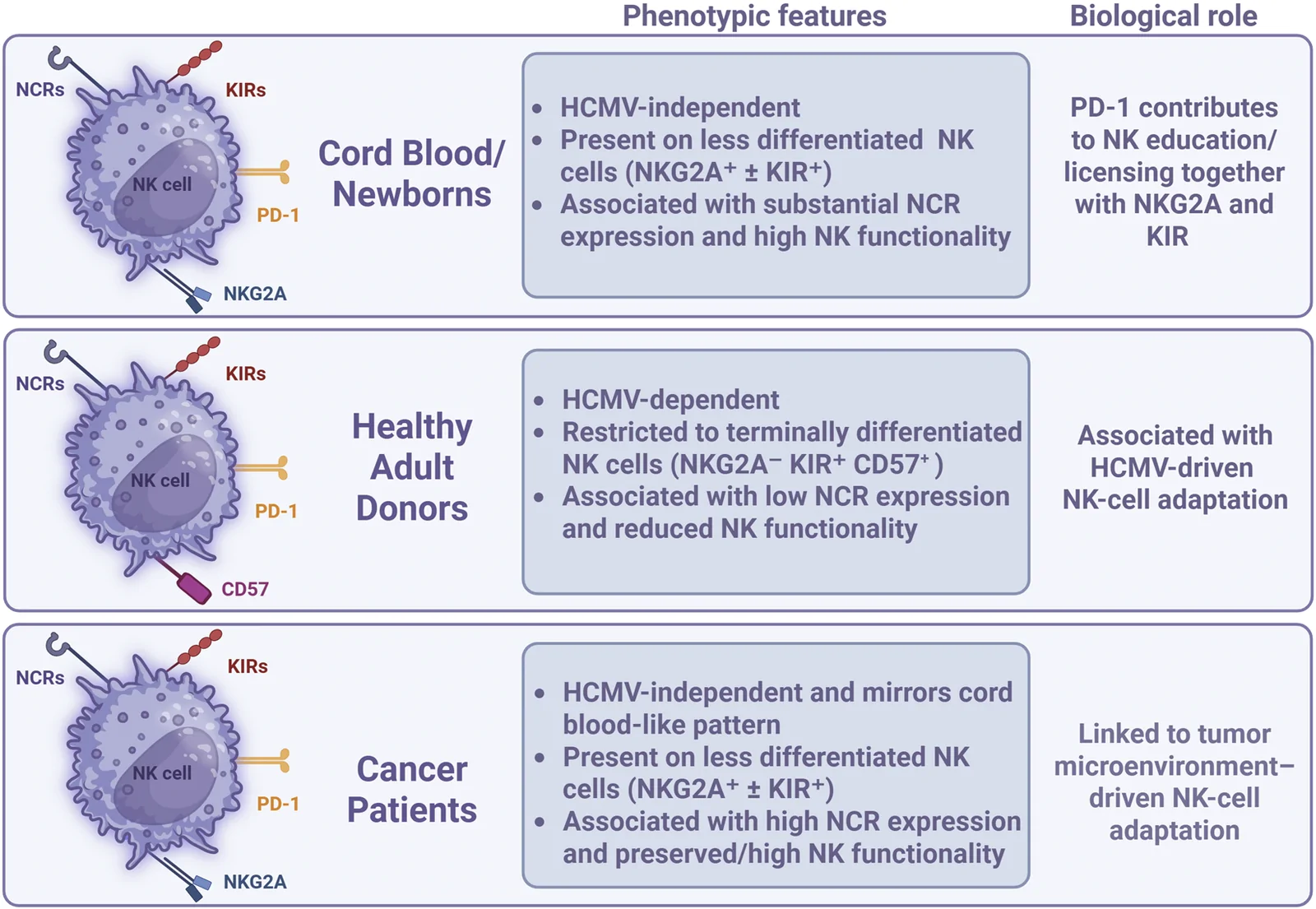
PD-1 expression patterns in cord blood/newborns, healthy adult donors, and cancer-associated NK cells. Cord blood/Newborns NK cells display higher levels of PD-1 compared to NK cells from healthy adult donors (HAD). In cord blood, PD-1 expression is HCMV-independent and is detected predominantly on less differentiated and highly functional NK cell subsets, characterized by NKG2A expression with or without KIR co-expression. These PD-1^+^ NK cells retain substantial expression of natural cytotoxicity receptors (NCRs) and, together with NKG2A and KIRs, contribute to NK cell education and licensing during development. In HAD, PD-1 expression is largely restricted to HCMV-driven, terminally differentiated NK cells (NKG2A- KIR+ CD57^+^). These cells typically exhibit reduced NCR expression and a hypofunctional phenotype, suggesting that PD-1 expression in this context is associated with diminished effector capacity. In cancer patients, PD-1 expression resembles the cord blood pattern rather than the healthy adult one. It is HCMV-independent and predominantly found on less differentiated NK cell subsets that can co-express NKG2A and KIRs. Unlike adult PD-1^+^ NK cells, these populations maintain high NCR expression and are generally highly functional, suggesting a context-dependent role of PD-1 within the tumor microenvironment.

PD-1 is not exclusively expressed on T lymphocytes but is also present on NK cells under both physiological and pathological conditions (Pesce et al., 2017; Greppi et al., 2023; Benson et al., 2010), where it functions as a key modulatory checkpoint of NK-cell activation and effector responses **(** Figure 1 **)**. In this context, PD-1 emerges as a context-dependent regulatory receptor that integrates differentiation status and environmental cues to fine-tune NK-cell responsiveness. Rather than representing a marker of terminal dysfunction, its expression is associated with a dynamic tuning of activation thresholds and preservation of cytotoxic potential.

These observations position PD-1 as a broader regulator of NK-cell biology beyond the T-cell compartment, highlighting its role in shaping immune responsiveness across different immunological settings.

### 3.1 Historical controversies surrounding PD-1 expression on NK cells

The identification of PD-1 expression on NK cells has long been a subject of debate. Unlike T lymphocytes, in which PD-1 is robustly induced upon activation, NK-cell expression is generally confined to specific subsets and is often detected at relatively low frequencies. These characteristics initially raised questions regarding the biological relevance of PD-1 in NK cells and contributed to conflicting observations across different studies.

Early reports describing PD-1-positive NK cells were primarily obtained in pathological settings. One of the first studies was conducted by Benson et al., who demonstrated PD-1 expression on NK cells from patients with multiple myeloma and showed that engagement of the PD-1/PD-L1 axis impaired NK-cell-mediated cytotoxicity against myeloma cells (Benson et al., 2010). Similar findings were subsequently reported in post-transplant lymphoproliferative disorders and other hematological malignancies (Wiesmayr et al., 2012). However, several groups failed to detect substantial PD-1 expression on circulating NK cells from healthy adult donors (HAD), raising doubts about the physiological relevance of this checkpoint receptor within the NK-cell compartment.

Several technical and biological factors likely contributed to these discrepancies. Variations in antibody clones, flow-cytometry gating strategies, donor selection criteria, and the distinction between intracellular and surface-associated PD-1 pools can all influence PD-1 detection. Moreover, because PD-1-positive NK cells generally represent only a minor fraction of circulating NK cells, these populations may have been overlooked in earlier studies (Vacca et al., 2019; Del et al., 2020).

A major advance came from the work of Pesce et al. (Pesce et al., 2017), who demonstrated that PD-1 expression was largely restricted to highly differentiated NK cells and strongly associated with HCMV seropositivity. These findings provided the first compelling evidence that endogenous PD-1 expression can occur in physiological conditions and is linked to NK-cell differentiation and adaptation to chronic immune stimulation (Pesce et al., 2017).

Importantly, PD-1 expression on NK cells is not limited to pre-existing differentiated subsets. Studies by Quatrini and colleagues demonstrated that PD-1 could be induced *de novo* in murine NK cells under inflammatory conditions through the combined action of glucocorticoids and pro-inflammatory cytokines, particularly IL-12, IL-15, and IL-18 (Quatrini et al., 2021; Quatrini et al., 2018). In models of viral infection and cancer, this inducible expression contributed to the functional restraint of NK-cell responses, suggesting that PD-1 acquisition can represent a dynamic adaptation to environmental cues rather than a fixed developmental feature.

More recently, additional mechanisms have been proposed to explain the presence of PD-1 on NK cells and to reconcile some of the discrepancies reported in the literature. Acquisition of PD-1 from neighboring cells through trogocytosis has been described in several tumor models. Using murine cancer models and human tumor samples, Hasim et al. demonstrated that NK cells can acquire PD-1 protein directly from PD-1-expressing tumor cells through membrane-transfer events, independently of endogenous PDCD1 transcription (Hasim et al., 2022). These findings indicate that not all PD-1 detected at the NK-cell surface necessarily derives from *de novo* gene expression and underscore the importance of distinguishing between transcriptionally regulated and externally acquired PD-1.

The biological significance of these observations has been extensively discussed in recent reviews. For example, Judge et al. emphasized that discrepancies among studies likely reflect both technical limitations and genuine biological heterogeneity across NK-cell subsets, disease settings, and tissue compartments (Judge et al., 2020). Collectively, these findings suggest that PD-1 biology in NK cells is more complex than initially anticipated and may involve differentiation-associated programs, cytokine-driven induction, and intercellular protein-transfer mechanisms.

Despite the historical controversies, substantial evidence now supports the existence of endogenous PDCD1 expression in specific NK-cell populations. PD-1-positive NK cells have been identified in highly differentiated HCMV-associated subsets (Pesce et al., 2017), neonatal NK cells (Greppi et al., 2023), and tumor-infiltrating NK cells in both hematological and solid malignancies (Pesce et al., 2017; Pesce et al., 2019; Benson et al., 2010; MacFarlane et al., 2014; Beldi-Ferchiou et al., 2016; Liu et al., 2017; Vari et al., 2018; Concha-Benavente et al., 2018; Trefny et al., 2020; Farhat et al., 2024; Tumino et al., 2019; Quatrini et al., 2021; Quatrini et al., 2018). Taken together, current evidence indicates that PD-1 represents a genuine component of NK-cell regulation. The remaining challenge is no longer to establish whether PD-1 can be detected in NK cells, but rather to define the cellular contexts, regulatory mechanisms, and relative contributions of endogenous expression versus protein acquisition that shape PD-1 biology in distinct physiological and pathological settings.

### 3.2 Immune checkpoints in healthy adult donor NK cells: from classical inhibitory receptors to PD-1

NK cells are innate lymphocytes that play a central role in immune surveillance by targeting virus-infected and transformed cells through direct cytotoxicity and cytokine secretion, thereby contributing to both innate and adaptive immune responses (Pesce et al., 2017; Hsu et al., 2018). Their activity is regulated by a dynamic balance between activating and inhibitory receptors, enabling the recognition and elimination of cells displaying the classical “missing self” phenotype associated with reduced HLA class I expression. Among activating receptors, the natural cytotoxicity receptors (NCRs) (Moretta et al., 2001), NKp30, NKp44, and NKp46, are key mediators of tumor and viral recognition. NKp30 interacts with ligands such as BAG6 and B7-H6 (Pesce et al., 2015; Pogge von Strandmann et al., 2007; Brandt et al., 2009), whereas NKp44 binds a variety of cellular ligands involved in immune recognition (Baychelier et al., 2013; Rajagopalan and Long, 2013). Additional activating receptors include NKG2D, which recognizes stress-induced ligands (MICA/B and ULBPs), DNAM-1, which binds CD112 and CD155, and the activating CD94/NKG2C heterodimer, which recognizes HLA-E and is preferentially expressed by adaptive-like NK-cell populations that expand and persist following HCMV infection (Pesce et al., 2017; Lopez-Vergès et al., 2011). Co-receptors such as 2B4 and NTBA further amplify activation signals and contribute to the fine-tuning of NK-cell responses (Sivori et al.; Bottino et al., 2001; Falco et al., 2004). In addition, CD16 (FcγRIIIa) mediates antibody-dependent cellular cytotoxicity (ADCC) by recognizing the Fc portion of IgG1-and IgG3-opsonized target cells (Vivier et al., 2011).

In contrast, inhibitory receptors, including killer immunoglobulin-like receptors (KIRs) and the inhibitory CD94/NKG2A heterodimer, recognize classical and non-classical HLA class I molecules, respectively, and transduce inhibitory signals that prevent autoreactivity. Notably, CD94/NKG2A and CD94/NKG2C share HLA-E as a ligand but exert opposing inhibitory and activating functions, respectively. The identification of KIRs, originally described as p58 molecules, by Alessandro Moretta and colleagues represented a landmark discovery in NK-cell biology, establishing the first conceptual framework for immune checkpoint regulation in NK cells (Pesce et al., 2019; Moretta et al., 2001; Sivori et al., 1996).

This paradigm ultimately paved the way for the development of NK-cell-directed checkpoint blockade strategies, including lirilumab (anti-KIR) and monalizumab (anti-NKG2A), aimed at restoring NK-cell cytotoxicity by functionally recreating “missing self” conditions (Pesce et al., 2019).

Within this expanding landscape of inhibitory receptors, PD-1 emerged as a particularly intriguing non-canonical checkpoint molecule in NK cells. Although initial reports described PD-1 expression primarily in pathological conditions, including multiple myeloma and post-transplant lymphoproliferative disorders (Benson et al., 2010; Wiesmayr et al., 2012), subsequent studies demonstrated that PD-1 is also present in healthy individuals. PD-1+ NK cells can be detected in approximately 20%–30% of HAD, although their frequency varies considerably between individuals (Pesce et al., 2017; Mariotti et al., 2019).

The molecular mechanisms operating downstream of PD-1 engagement in NK cells remain less comprehensively characterized than those described in T lymphocytes. Available evidence suggests that PD-1 signaling interferes with pathways involved in cytotoxicity, cytokine production, and cellular metabolism, ultimately promoting a functionally restrained phenotype (Pesce et al., 2017; Judge et al., 2020; Hsu et al., 2018).

Phenotypically, PD-1 expression is largely restricted to a subset of highly differentiated CD56^dim^ NK cells characterized by a NKG2A- KIR+ CD57^+^ phenotype, high expression of CD16, perforin, and granzyme, and reduced levels of NCRs such as NKp30 and NKp46, suggesting a link with terminal differentiation and adaptive-like NK features rather than conventional exhaustion (Pesce et al., 2017) (Figure 1).

Functionally, these cells display impaired natural cytotoxicity, reduced degranulation, diminished responsiveness to cytokines such as IL-2, IL-12, IL-15, and IL-18, partly attributable to decreased CD122 expression, and impaired cytokine production in response to target cells. Nevertheless, their cytotoxic machinery remains largely preserved, as evidenced by intact CD16-mediated responses, suggesting that PD-1 primarily raises the threshold for NK-cell activation rather than driving terminal functional exhaustion or irreversible loss of effector function (Pesce et al., 2017). Importantly, a strong association has been established between the presence of PD-1+ NK cells and HCMV seropositivity. Virtually all HAD harboring detectable PD-1+ NK-cell populations were found to be HCMV-seropositive (Pesce et al., 2017), suggesting that chronic viral exposure plays a critical role in shaping this subset. HCMV infection is known to drive the expansion of adaptive-like NK cells characterized by NKG2C expression and extensive epigenetic remodeling. However, PD-1+ NK cells only partially overlap with this population.

Indeed, although these cells may co-express NKG2C, their phenotype remains distinct from that of classical adaptive NK cells, which typically display Siglec-7 downregulation and a more restricted receptor repertoire. Rather than defining a purely HCMV-driven adaptive subset, PD-1 expression appears to identify a broader trajectory of terminal NK-cell differentiation associated with chronic immune stimulation.

Collectively, these features support the notion that PD-1 marks a highly differentiated and functionally restrained NK-cell compartment, potentially serving as a physiological mechanism to limit excessive immune activation during persistent antigenic stimulation.

### 3.3 Identification of PD-1+ natural killer cells in neonates

The identification of PD-1-expressing NK cells in cord blood provided important insights into the biology of this checkpoint receptor and challenged the prevailing view that PD-1 expression is exclusively associated with chronic antigenic stimulation or advanced NK-cell differentiation. Indeed, the detection of PD-1 on neonatal NK cells demonstrated that checkpoint receptor acquisition can occur early during NK-cell ontogeny and may represent a physiological component of NK-cell development (Greppi et al., 2023).

In contrast to adult peripheral blood, where PD-1 expression is largely confined to highly differentiated NK-cell subsets, neonatal PD-1+ NK cells display an immature-to-intermediate phenotype. In cord blood, PD-1 is predominantly expressed within the CD56^dim^ and CD56^neg^CD16+ compartments, whereas it is largely absent from CD56^bright^ NK cells. These cells frequently co-express NKG2A and KIRs, consistent with an intermediate stage of NK-cell maturation. Importantly, PD-1 expression is not detected in CD34^+^ hematopoietic progenitors, indicating that its acquisition occurs during NK-cell differentiation rather than at the stem-cell stage (Greppi et al., 2023).

A major distinction from adult PD-1+ NK cells is that neonatal PD-1 expression develops independently of HCMV exposure. Because cord blood is derived from immunologically naïve individuals, these findings demonstrate that PD-1 acquisition does not require chronic viral infection or prolonged antigenic stimulation. Instead, they support the concept that PD-1 expression can be developmentally programmed and integrated into the physiological maturation process of NK cells (Figure 1).

Functional analyses further revealed that PD-1 expressed by cord blood NK cells is fully competent and capable of transducing inhibitory signals upon engagement by PD-L1 or PD-L2. Ligation of PD-1 results in reduced degranulation and diminished IFN-γ production, confirming its role as an active inhibitory receptor. Notably, however, in the absence of PD-1 engagement, PD-1+ NK cells, particularly those lacking both KIRs and NKG2A, can exhibit enhanced cytotoxicity and cytokine production compared with their PD-1- counterparts. These observations suggest that PD-1 functions as a tunable regulatory checkpoint that modulates activation thresholds rather than simply marking functionally impaired cells.

Consistent with this interpretation, neonatal PD-1+ NK cells exhibit a distinct pattern of NCR expression. Although NCRs are generally reduced on PD-1+ cord blood NK cells compared with their PD-1- counterparts, both NKp46 and NKp30 are upregulated in PD-1+ cord blood NK cells relative to HAD NK cells, with this effect being particularly evident within the PD-1+ CD56^dim^ subset. Together, these findings suggest a nuanced modulation of NK-cell maturation rather than a uniform suppression of NCR expression (Figure 1).

This profile supports the hypothesis that PD-1 signaling may contribute to the progressive acquisition of functional competence by preventing premature or excessive activation. This model aligns with the broader concept of NK-cell education, whereby inhibitory signals shape NK-cell responsiveness to ensure self-tolerance while preserving future effector potential.

The comparison between neonatal and adult PD-1+ NK cells highlights important biological differences. Adult PD-1+ NK cells are predominantly associated with terminal differentiation, chronic immune stimulation, and HCMV seropositivity, and generally exhibit a more restrained functional profile. In contrast, neonatal PD-1+ NK cells are developmentally immature, arise independently of chronic antigen exposure, and retain substantial functional plasticity. These observations suggest that PD-1 fulfills distinct biological roles depending on the developmental and immunological context in which it is expressed (Pesce et al., 2017; Greppi et al., 2023).

Collectively, these findings support a broader view of PD-1 biology in NK cells. Rather than representing solely a marker of chronic stimulation or dysfunction, PD-1 appears to participate in the physiological regulation of NK-cell maturation and functional calibration from the earliest stages of development. This concept has important implications for both basic immunology and clinical translation. In particular, the increasing use of cord blood-derived NK cells in adoptive immunotherapy raises the possibility that modulation of the PD-1 pathway could influence NK-cell maturation, persistence, and antitumor activity. More broadly, the identification of PD-1+ NK cells in neonates underscores the context-dependent nature of checkpoint receptor biology and suggests that PD-1 may regulate distinct developmental, homeostatic, and pathological programs throughout the NK-cell life cycle.

## 4 PD-1+ NK cells in cancer

In cancer, NK cells play a central role in tumor immunosurveillance due to their ability to recognize transformed cells, independently of antigen presentation, through “missing self” recognition. Across both hematological and solid malignancies, NK-cell function is frequently altered by chronic exposure to tumor-derived inhibitory signals and suppressive microenvironmental cues.

PD-1 is expressed on NK cells in a variety of hematological and solid malignancies and is associated with impaired cytotoxicity, reduced cytokine production, and compromised immune surveillance (Pesce et al., 2017; Pesce et al., 2019; Benson et al., 2010; MacFarlane et al., 2014; Beldi-Ferchiou et al., 2016; Liu et al., 2017; Vari et al., 2018; Concha-Benavente et al., 2018; Trefny et al., 2020; Farhat et al., 2024; Tumino et al., 2019; Quatrini et al., 2021; Quatrini et al., 2018).

The clinical relevance of this immune checkpoint was underscored by the rapid development and clinical implementation of PD-1/PD-L1 blockade therapies. Pivotal phase I trials with nivolumab (BMS-936558) and subsequent studies with pembrolizumab demonstrated durable clinical responses in melanoma, non-small cell lung cancer, and renal cell carcinoma (Topalian et al., 2012; Weber et al., 2015; Robert et al., 2015). Although initially conceived as T-cell reinvigoration strategies, it is now evident that their therapeutic efficacy also involves innate immune compartments, particularly NK cells, which are essential effectors in tumors with low or absent HLA class I expression and limited neoantigen availability, thereby escaping effective CD8^+^ T-cell recognition (André et al., 2018; Hsu et al., 2018).

NK cells do not appear to fully recapitulate the canonical exhaustion programs described in T lymphocytes. The concept of exhaustion was originally defined in chronically stimulated T cells and is characterized by progressive loss of effector function, sustained expression of inhibitory receptors, profound transcriptional remodeling, and stable epigenetic reprogramming (Blank et al., 2019; Myers et al., 2022).

Whether NK cells undergo an analogous process remains an area of active investigation. As observed in T cells, chronically stimulated NK cells can upregulate inhibitory receptors in settings of persistent activation (Pesce et al., 2017; Benson et al., 2010; André et al., 2018; Zhang et al., 2018; Da Silva et al., 2014; Blake et al., 2016; Seo et al., 2017; Van Hall et al., 2019); however, accumulating evidence indicates that they often retain substantial cytotoxic potential and can rapidly restore effector functions upon appropriate stimulation or relief from inhibition, underscoring their marked functional plasticity (Myers et al., 2022; Judge et al., 2020). Consequently, alternative concepts such as NK-cell adaptation, checkpoint-restrained states, or dysfunctional differentiation have been proposed. These models suggest that chronic exposure to tumor-derived factors, inflammatory mediators, and metabolic stress progressively remodels NK-cell responsiveness without necessarily inducing the terminal epigenetic programs characteristic of exhausted T cells (Blank et al., 2019). This distinction has important therapeutic implications, as the potentially reversible nature of NK-cell dysfunction may render these cells particularly attractive targets for checkpoint blockade and cell-engineering approaches (Myers et al., 2022).

In addition to regulating natural cytotoxicity, PD-1 may also influence CD16-mediated NK-cell effector functions. However, evidence supporting a direct role of the PD-1/PD-L1 axis in the regulation of ADCC remains limited. One of the few studies specifically addressing this question demonstrated that PD-1 blockade enhances CD16-dependent NK-cell degranulation, cytokine production, and cytotoxicity against PD-L1-expressing, antibody-opsonized tumor cells, suggesting that PD-1 signaling may restrain FcγRIIIa-mediated activation under these conditions (Siebert et al., 2017). These observations suggest that PD-1 inhibition may enhance the efficacy of antibody-based cancer immunotherapies by potentiating Fc receptor-dependent NK-cell functions. However, whether PD-1 directly interferes with CD16 signaling or instead acts by increasing the activation threshold of NK cells remains unclear, and the molecular mechanisms linking PD-1 signaling to FcγRIIIa-mediated activation require further investigation.

Importantly, immune checkpoints are not only intrinsic regulators of NK-cell activity but also modulate their cross-talk with other immune populations, including dendritic cells and T cells. Through these interactions, NK cells can influence dendritic cell maturation and T-cell priming, thereby shaping the quality and magnitude of adaptive immune responses and positioning themselves at the interface between innate and adaptive immunity (Marcenaro et al., 2005; Morvan and Lanier, 2016; Cichocki et al., 2020; Barry et al., 2018; Böttcher et al., 2018). Accordingly, immune checkpoint blockade should be viewed as a system-level intervention that acts on interconnected immune networks rather than on isolated cellular targets, ultimately reshaping both innate and adaptive immune responses in a coordinated manner.

Early studies in multiple myeloma and post-transplant lymphoproliferative disorders demonstrated that PD-1 expression on NK cells is associated with impaired cytotoxicity and reduced tumor control, supporting a direct role for this pathway in NK-cell dysfunction and immune evasion (Benson et al., 2010; Wiesmayr et al., 2012).

PD-1-expressing NK cells have also been described in lymphoma and other lymphoid malignancies, where chronic exposure to tumor-derived inhibitory signals promotes the acquisition of a dysfunctional phenotype characterized by reduced effector function and co-expression of additional inhibitory receptors (Beldi-Ferchiou et al., 2016; Vari et al., 2018).

In classical Hodgkin lymphoma (cHL), Reed-Sternberg (RS) cells exhibit constitutive PD-L1 overexpression driven by 9p24.1 amplification (Green et al., 2010), rendering this disease highly sensitive to PD-1 blockade. Notably, RS cells are also characterized by loss or marked downregulation of HLA class I expression, a feature that further increases their susceptibility to NK-cell-mediated immune surveillance by limiting effective T-cell recognition while creating a permissive context for innate immune recognition. Based on the clinical efficacy of PD-1 inhibition, in 2017, nivolumab was approved for the treatment of relapsed or refractory Hodgkin lymphoma following hematopoietic stem cell transplantation (Ansell, 2015; Kasamon et al., 2017).

Beyond restoring T-cell function, clinical responses to nivolumab have also been associated with profound remodeling of the NK-cell compartment. PD-1 blockade promotes the expansion of highly differentiated NK-cell populations, including CD56^neg^CD16^bright^ subsets and adaptive NKG2C + NK cells, suggesting that PD-1 blockade indirectly reshapes NK-cell differentiation and activation programs (Guolo et al., 2021). These expanded NK-cell populations can contribute not only to direct tumor cell elimination but also to long-term immunosurveillance, limiting subsequent tumor growth and further supporting the therapeutic value of PD-1 blockade in cHL, and potentially additional malignancies (Guolo et al., 2021).

These findings provide a mechanistic perspective in which PD-1 blockade emerges not only as a strategy to reinvigorate T-cell responses, but also as a broader immunomodulatory intervention capable of reshaping NK-cell differentiation and sustaining innate immune surveillance in a clinically relevant manner.

In solid tumors, PD-1+ NK cells emerge as key components of tumor-infiltrating dysfunctional immune states, with context-dependent features shaped by tumor immunogenicity and microenvironmental organization (Paleari et al., 2021; Pesce et al., 2020; Greppi et al., 2019).

In colorectal cancer, PD-1–expressing NK cells show a clear association with microsatellite instability (MSI) status. MSI-high tumors are enriched in tumor-infiltrating NK-cell populations expressing PD-1, alongside other checkpoint receptors, and these cells often display features of tissue adaptation such as CD103 and CD49a expression, consistent with a tumor microenvironment-driven reprogramming and retention within the tissue (Obino et al., 2025). Importantly, these PD-1+ NK cells retain a partially cytotoxic and activated phenotype, suggesting they are not fully exhausted but instead functionally reshaped by the MSI-associated inflammatory milieu.

In contrast, microsatellite-stable (MSS) tumors generally show lower immune infiltration and a less prominent PD-1+ NK-cell compartment. In this setting, NK-cell activity appears more constrained by a combination of inhibitory pathways, including NKG2A, and KIR signaling, together with preserved HLA class I expression on tumor cells, which collectively contributes to stronger restraint of NK-cell effector functions.

Triple-negative breast cancer (TNBC) represents another clinically relevant setting in which NK-cell checkpoints contribute to immune escape (Park et al., 2017). Although PD-1/PD-L1 blockade combined with chemotherapy improves outcomes in PD-L1+ disease, overall response rates remain limited, reflecting both intrinsic and acquired resistance mechanisms within a highly immunosuppressive TME (Rebaudi et al., 2024; Geurts and Kok, 2023).

Recent evidence further refines the role of NK cells in this context, showing that tumor-derived IL-18 can directly drive the upregulation of PD-1 on NK cells in TNBC, promoting the emergence of an immunosuppressive NK-cell subset with impaired effector function (Park et al., 2017). This mechanism links inflammatory signaling within the TME to checkpoint acquisition on NK cells, positioning the IL-18-PD-1 axis as a key regulator of NK-cell dysfunction in TNBC.

Spatial immune profiling further supports the existence of compartmentalized immune suppression within tumors, with discrete regions enriched in inhibitory ligands, stromal cues, and myeloid populations that collectively reinforce NK-cell impairment and may sustain PD-1-mediated dysfunction (Keren et al., 2018).

Ovarian cancer, particularly high-grade serous carcinoma (HGSC), represents one of the most striking models of NK-cell checkpoint dysregulation and immune reprogramming within the TME (Pesce et al., 2017; Pesce et al., 2019; Greppi et al., 2019). In this setting, PD-1+ NK cells are not a marginal subset but a dominant feature of the immune landscape, being enriched in both peripheral blood and, even more prominently, in ascitic fluid, where they are exposed to sustained tumor-derived inhibitory signals. Functionally, these cells exhibit a clear reduction in cytotoxicity and cytokine production upon engagement with PD-L1/PD-L2-expressing targets, with evidence that PD-1 blockade can partially restore their effector functions, underscoring the therapeutic relevance of this axis.

Importantly, recent evidence by Greppi et al. (Greppi et al., 2025) has radically reframed the biological significance of PD-1 expression on NK cells, highlighting its strong context dependency. Indeed, PD-1+ NK cells are relatively uncommon under physiological conditions, being detected in approximately 25% of HAD and around 51% of neonates, whereas their frequency increases dramatically in ovarian cancer, reaching approximately 93% of patients. This sharp gradient underscores PD-1 as a hallmark of tumor-driven NK-cell reprogramming rather than a constitutive feature of NK-cell biology, reflecting the profound impact of the ovarian TME on shaping inhibitory receptor landscapes. Notably, a substantial proportion of PD-1+ NK cells display a tissue-resident phenotype, suggesting that local tissue-specific signals contribute to the acquisition and maintenance of PD-1 expression. Moreover, PD-1 expression progressively increases along disease evolution, from the primary tumor to ascitic fluid and ultimately to metastatic lesions, indicating a close association with tumor progression. Consistent with this observation, higher frequencies of PD-1+ NK cells correlate with poor clinical outcome, further supporting the relevance of this inhibitory receptor as a marker of disease aggressiveness.

Within this immunosuppressive milieu, the most prominent and functionally relevant NK-cell population is represented by PD-1+NKG2A + double-positive cells, which constitute the dominant subset within tumor sites (Figure 1). This co-expression defines a convergent inhibitory program in which two non-redundant checkpoints synergize to dampen NK-cell effector responses: PD-1 mediates suppression through the PD-L1 axis, while NKG2A engages HLA-E-dependent inhibition. The enrichment of this double-positive subset in HGSC provides a mechanistic explanation for the profound yet potentially reversible immune dysfunction observed in these patients.

**FIGURE 2:**
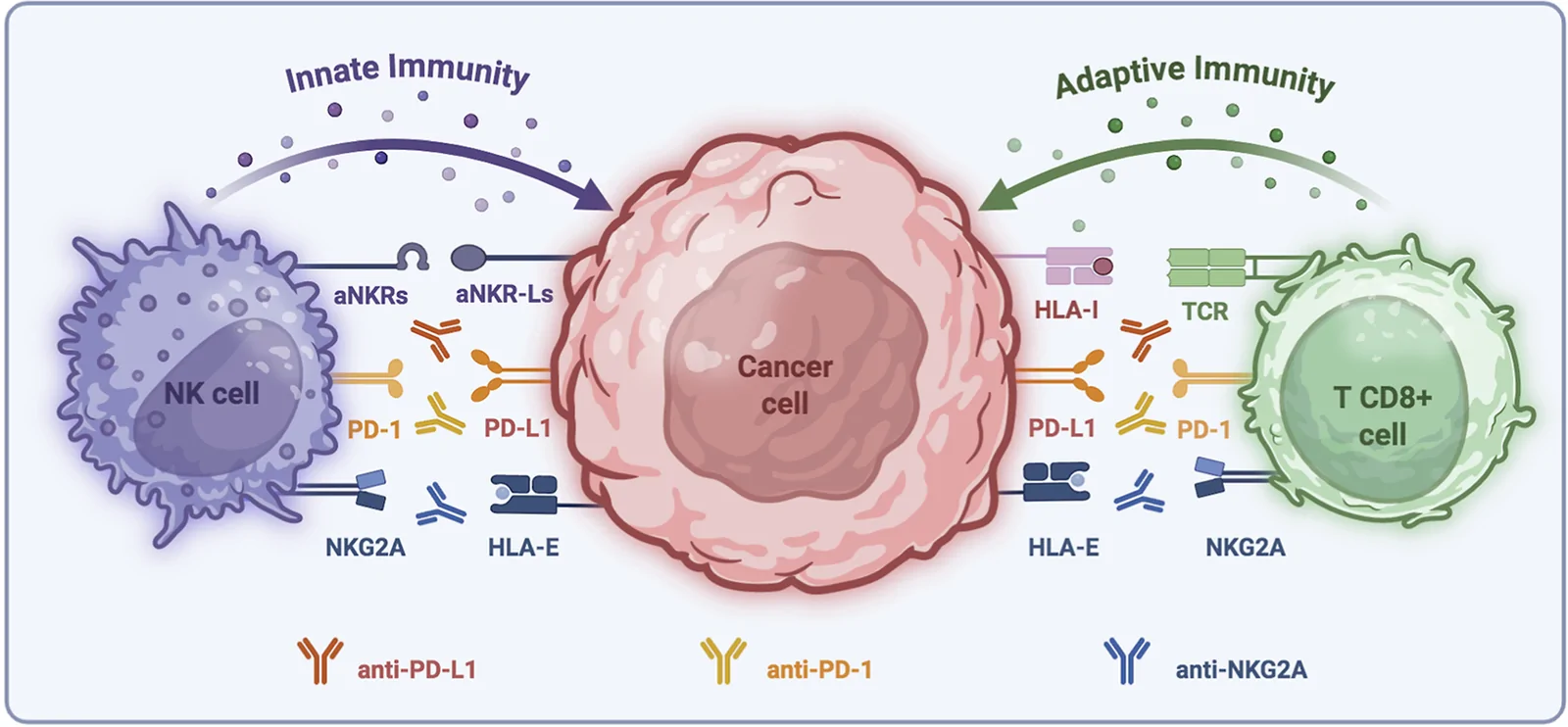
Schematic representation of innate and adaptive antitumor immunity and their modulation by immune checkpoint blockade. Natural killer (NK) cells and T lymphocytes are key mediators of innate and adaptive immunity, respectively, exerting antitumor effects through cytotoxic granule release and tumor-cell recognition mediated by NK activating receptors (aNKRs) and antigen-specific T-cell receptor (TCR). Tumor cells express ligands for NK-cell activating receptors (aNKR-Ls) as well as inhibitory molecules, including PD-L1 and HLA-E. PD-1 is expressed on both NK and T cells and, upon interaction with PD-L1 on tumor cells, suppresses effector functions and promotes immune evasion. Similarly, engagement of NKG2A on NK and T cells by HLA-E delivers inhibitory signals that further restrain antitumor activity. Therapeutic blockade of these pathways using anti-PD-1, anti-PD-L1, and anti-NKG2A monoclonal antibodies restores cytotoxic responses and enhances both innate and adaptive antitumor immunity.

These findings have direct translational implications. The dominance of PD-1+NKG2A + NK cells within the TME strongly supports a rational, mechanism-driven therapeutic strategy based on combined checkpoint blockade targeting both PD-1 and NKG2A. Such a dual approach is likely to be more effective than single-axis inhibition, as it simultaneously dismantles the principal inhibitory circuits converging on NK-cell function, thereby offering a more precise and potent means to restore anti-tumor immunity in ovarian cancer patients (Figure 2).

Recent advances in single-cell transcriptomics and high-dimensional immune profiling have substantially refined our understanding of NK-cell heterogeneity within tumors (Crinier et al., 2018; Dogra et al., 2020). Rather than representing a homogeneous population, tumor-infiltrating NK cells comprise multiple transcriptional and functional states shaped by tissue residency, differentiation, inhibitory receptor expression, and local microenvironmental cues, highlighting the remarkable diversity of NK-cell responses across tumor types and tissue niches (Tang et al., 2023; Netskar et al., 2024). Accordingly, NK-cell dysfunction is now viewed as the result of coordinated transcriptional and phenotypic programs driven by inhibitory receptor signaling together with metabolic stress, cytokine exposure, tissue localization, and interactions with stromal and myeloid cells, rather than by any single checkpoint pathway (Crinier et al., 2021; Hu et al., 2021; Li and O’Sullivan, 2022; Rishabh and Matosevic, 2025).

A key determinant of these dysfunctional states is metabolic reprogramming. NK-cell activation normally requires increased glycolysis, oxidative phosphorylation, and nutrient uptake to sustain cytotoxicity and cytokine production (Michelet et al., 2018; O’B et al., 2019). However, within the TME, nutrient deprivation, hypoxia, lactate accumulation, TGF-β, and other suppressive factors impair mTOR signaling, glycolytic metabolism, mitochondrial fitness, and ultimately effector function (Michelet et al., 2018; Terrén et al., 2019; Sohn and Cooper, 2023; Cózar et al., 2021; Viel et al., 2025; Ielpo et al., 2026), that appear to be partially reversible, supporting the concept of functional plasticity in dysfunctional NK cells. Although PD-1 expression has been reported in subsets of tumor-infiltrating NK cells, current evidence indicates that their dysfunctional phenotype primarily reflects the combined impact of inhibitory receptor networks and metabolic constraints rather than PD-1 signaling alone.

Collectively, these data support a model in which NK-cell dysfunction arises from the integration of multiple inhibitory pathways rather than PD-1 signaling alone, providing a rationale for combinatorial checkpoint targeting strategies, as well as for the combined use of PD-1-axis blockade and metabolic interventions to enhance NK-cell persistence and antitumor activity.

## 5 Discussion

The growing recognition that PD-1 is functionally expressed on NK cells, often in combination with additional inhibitory receptors such as NKG2A, has substantially broadened the conceptual framework of immune checkpoint biology beyond adaptive immunity. Although PD-1/PD-L1 blockade was initially designed to reinvigorate exhausted T cells, accumulating evidence indicates that NK cells also represent both direct targets and key effectors of these therapies, particularly in tumors characterized by low HLA class I expression or poor T-cell infiltration (André et al., 2018; Hsu et al., 2018).

A central concept emerging from recent studies is that PD-1 does not define a uniform NK-cell state. In healthy adults, its expression is largely confined to HCMV-associated terminally differentiated NK cells, whereas in neonates and cancer patients it is more frequently detected in less differentiated NKG2A + subsets that retain substantial functional competence. This context dependency highlights PD-1 as a dynamic marker shaped by NK-cell differentiation trajectories and microenvironmental cues, rather than a fixed feature of exhaustion.

Across both hematological and solid malignancies, PD-1 operates within integrated inhibitory circuits rather than in isolation. Ovarian cancer exemplifies this principle, where PD-1+ NK cells localize within PD-L1+/HLA-E+ suppressive niches and frequently co-express NKG2A, defining a coordinated checkpoint landscape that reinforces functional restraint (Greppi et al., 2025). More broadly, these observations support a model in which NK-cell dysfunction arises from the convergence of multiple inhibitory pathways within spatially organized TMEs.

From a therapeutic standpoint, this framework provides a rationale for both single- and multi-axis checkpoint targeting. While PD-1/PD-L1 blockade can restore NK-cell function in settings where PD-1+ NK cells retain activating receptor expression, growing evidence suggests that monotherapy is unlikely to fully overcome the layered inhibitory networks operating in most tumors. Accordingly, combinatorial strategies targeting complementary checkpoints, together with emerging NK-cell engineering approaches, are gaining traction as more effective means to circumvent immune suppression (Cichocki et al., 2020; Susek et al., 2023).

Importantly, the shared utilization of checkpoints such as PD-1 and NKG2A by NK and T cells underscores the broader immunological reach of checkpoint blockade, extending its impact beyond adaptive immunity to coordinate innate and adaptive antitumor responses. This dual engagement may partly explain the clinical heterogeneity of responses and suggests additional layers of therapeutic opportunity (Figure 2).

Future efforts should move beyond static phenotyping toward integrated single-cell, spatial, and functional dissection of NK-cell states in human tumors. Defining how checkpoint pathways intersect with NK-cell differentiation, metabolic fitness, and tissue-specific adaptation will be critical for the rational design of next-generation immunotherapies, including combinatorial checkpoint blockade and engineered NK-cell platforms tailored to patient-specific immune landscapes.
